# Circ_0039908/miR-let-7c/RRM2 axis was identified played an important role in lung adenocarcinoma by integrated analysis

**DOI:** 10.7150/jca.72789

**Published:** 2022-07-18

**Authors:** Weijie Zhang, Jun Chen, Yue Li, Ruochen Zhang, Anqi Wang, Yuanyuan Zeng, Jianjie Zhu, Zeyi Liu, Jian-an Huang

**Affiliations:** 1 Department of Pulmonary and Critical Care Medicine, the First Affiliated Hospital of Soochow University, Suzhou, 215006, China.; 2 Suzhou Key Laboratory for Respiratory Diseases, Suzhou, 215006, China.; 3 Institute of Respiratory Diseases, Soochow University, Suzhou, 215006, China.; 4 Department of Thoracic Surgery, The First Affiliated Hospital of Soochow University, Medical College of Soochow University, Suzhou, China.

**Keywords:** Lung adenocarcinoma, circRNA, competitive endogenous RNA, bioinformatics analysis

## Abstract

**Background:** Circular RNAs (circRNAs) are shown to play a significant role in cancer initiation and progression by interacting on microRNAs (miRNAs) which act as one kind of competing endogenous RNAs (ceRNAs) for the regulation effect on target gene expressions. This study was performed to explore the prognosis-related circRNAs in lung adenocarcinoma (LUAD) patients by integrated analysis and find the mechanism it worked.

**Methods:** The miRNAs and mRNAs, accompanied with circRNAs expressions were obtained through The Cancer Genome Atlas (TCGA) and the Gene Expression Omnibus (GEO) database, The cytoHubba app of Cytoscape was used to identify hubgenes. Quantitative real-time PCR (q-RT PCR) was performed to identify the expression of circRNA, miRNA and mRNA, Cell Counting Kit-8 (CCK-8) and clone formation assays were used to evaluate the proliferation ability of different kinds of cells in vitro. Transwell assays were utilized to assess the motility of tumor cells.

**Results:** Finally, circRNA_0039908/let7c-5p/RRM2 axis was identified in our research, it can play an important role in the LUAD pathogenesis progression and we found that the proliferation, invasion and migration abilities of LUAD cells can be suppressed after knockdown of circRNA_0039908. This work indicates that circRNA_0039908/let7c-5p/RRM2 axis may be a promising target in the prognosis and treatment of LUAD patients.

**Conclusions:** Circ_0039908/miR-let-7c/RRM2 axis can promote the ability of proliferation, migration and invasion of LUAD cells.

## Introduction

As a kind of malignant tumor, lung carcinoma exhibits the highest velocity of mortality and morbidity growth, with the predictions of newly increased patients and fatality as 2.1 million and 1.8 million respectively in 2018 1, which enormously threatens the healthy life of human beings. Among the common subtypes of lung cancer, non-small cell lung cancer (NSCLC) represents 85% of lung cancer cases 2 and lung adenocarcinoma (LUAD) is categorized as the most common histological type 3. Despite the recent efforts in improvements in early diagnosis and treatment have been made, the prognosis of patients with LUAD was still unsatisfactory 4. Consequently, it is of crucial significance to further explore the mechanism of occurrence and metastasis of LUAD, and to provide new ideas for improving treatment methods and exploring new therapeutic targets.

Unlike protein-coding RNAs, non-coding RNAs do not code for proteins and were once thought to be junk RNAs. But recent studies have shown that non-coding RNAs are key regulators of many important biological processes 5. Circular RNAs (circRNAs) are a special class of non-coding RNAs with covalent closed - loop structure without 5′ caps or 3′ tails which likely confer high resistance to RNA exonuclease or RNase R activity conferring much higher stability than linear RNAs 6. CircRNAs have been shown in many studies to be associated with physiological processes and development of various malignant tumors including lung cancer 7, 8, Recent studies have demonstrated that circRNAs can act as miRNA sponges, thereby inhibiting miRNA activity and further regulating the expression of their downstream target genes. For example, circRNA8924 promotes proliferation, migration, and invasion of cervical cancer cells by competitively binding the mir-518d-5p/519-5p family and modulating the expression of CBX8 9. As for the oral squamous cell carcinoma, the role that circRNA_100290 exerts is a kind of ceRNA that counteracts the GLUT1 inhibition induced by miR‐378a, thereby increasing the proliferating and glycolytic activity 10.

In this study, we successfully constructed a ceRNA regulatory network that contains mRNA, miRNA and circRNA, as well as a sub-network of circRNA-miRNA-hubgene. Finally, we found that the proliferation invasion and migration abilities of LUAD cells can be suppressed after knockdown of circRNA_0039908, while circRNA_0039908/miR-let7c/RRM2 axis was identified in our research.

## Methods and Materials

### Data collection

We screened the expression spectrum of circRNA in Gene Expression Omnibus (GEO) datasets (http://www.ncbi.nlm.nih.gov/gds/) until October 2020. We screened out the GSE101684(GPL21825, Agilent-074301 Arraystar Human CircRNA microarray V2, Agilent Technologies Inc, California, USA) dataset, which included 4 normal lung tissues and 4 LUAD tissues. Meanwhile, we downloaded miRNAseq and mRNAseq data of LUAD from the TCGA database by means of the Data Transfer Tool (obtained from GDC Apps) (https://tcga-data.nci.nih.gov/) with the closing date of 30 October 2020. The miRNA profiles contained 483 LUAD tissue samples and 45 nearby healthy lung tissue samples, and the profiles of mRNAs contained 497 LUAD tissues and 54 adjacent normal tissues. This study did not require ethical approval or informed consent since we used public data from GEO and TCGA.

### Differential expression analysis

The procession of download files including data calibration, normalization, and log2 conversion was conducted by means of R packages. The screening of circRNAs with differential expressions (DEcircRNAs) was performed utilizing the Limma package, and the criteria included adjusted P-value < 0.05 accompanied with |log 2 (fold change [FC])| > 1.5. In addition, the screening of miRNAs and mRNAs with differential expressions (DEmiRNAs and DEmRNAs, respectively) was performed utilizing the edgeR package, and the threshold values included adjusted P-value < 0.05 accompanied with |log 2 (fold change [FC])| > 1. Hierarchical clustering analysis of DEcircRNAs was performed using R package “heatmap” and volcano plots of DEmRNAs and DEmiRNAs were drawn using R package “gplots”.

### Construction of the competing endogenous RNA regulatory network

The prediction of miRNA response elements (MREs) and corresponding target miRNAs was conducted using the Cancer Specific CircRNA database (http://gb.whu.edu.cn/CSCD/). Only miRNAs that presented in both target miRNAs and DEmiRNAs on the basis of the TCGA database were filtered for network construction. Next, three databases (miRDB, miRTarBase and TargetScan) were applied to the prediction of the target mRNAs of DEmiRNAs. The precondition of being selected as mRNA candidates was the recognition of all three databases. The intersection between the candidate mRNAs and DEmRNAs was subsequently performed for screening the target DEmRNAs of DEmiRNAs, and the overlapping mRNAs were retained for constructing the network of ceRNAs. The pairs of miRNA-mRNA and circRNA-miRNA were combined to construct the regulation network of circRNA-miRNA-mRNA. Ultimately, Cytoscape v3.7.2 (http://www.cytoscape.org) was used to visualize the network.

### PPI network establishment and module analysis

For the assessment of the interaction among DEmRNA varieties, a PPI network was established by means of the Search Tool for the Retrieval of Interacting Genes (STRING) online tool (STRINGdb: https://string-db.org/), which can provide comprehensive interactions between proteins and genes. The PPI network was constructed with a combined score of > 0.9 as the screening criterion. The cytoHubba app was then used to extract hubgenes from the PPI network, which was visualized by Cytoscape.

### Prognostic analysis

To evaluate the effect of the expression level of hubgenes on the overall survival of LUAD patients, we entered the 6 miRNAs and 10 hubgenes into the Kaplan-Meier Plotter website to draw the Kaplan Meier curve. Log-rank test was then carried out and p< 0.05 was considered significant.

### Patient samples

A total of 14 paired LUAD tissue specimens were collected in the Department of Pulmonary and Critical Care Medicine, First Affiliated Hospital of Soochow University.The detailed clinic parameters of enrolled patients were listed in Table 3. All participants were provided with written informed consent at the time of recruitment. All samples were kept at -80 °C for storage. All cases had clinically and pathologically confirmed diagnoses of NSCLC based on the Revised International System for Staging Lung Cancer. The present study was approved by the Ethics Committee of the First Affiliated Hospital of Soochow University.

### Cells and culture

A549, H1299 and BEAS-2B (human immortalized normal epithelial cells) cells were purchased from the Cell Bank of the Chinese Academy of Sciences (Shanghai, China). Cells were cultured in RPMI 1640 medium and DMEM with 10% foetal bovine serum (Gibco, Carlsbad, CA, USA) and L-glutamine (Invitrogen, Carlsbad, CA, USA) at 37 °C in a 5% CO_2_ atmosphere.

### RNA extraction, cDNA synthesis and quantitative real-time PCR analysis

The detailed processes were performed as we previously described 11. Primers used in the study were listed in Table 1. The primer of miR-let-7c was designed by Accurate biology, and mRQ3' primer was supplied with the kit. The CT values of the gene mRNA levels were normalized to those of β-actin and U6. The ^△△^Ct method was applied to calculate the relative quantities of these mRNAs. Each experiment was performed in triplicate.

### Western blot analysis

Western blot analysis was performed as we previously described 12. The following antibodies were used in the analysis: anti-Akt, anti-p-Akt (Ser473), anti-ERK, anti-p-ERK (Thr202/Tyr204) (Cell Signaling Technology), anti-N-cadherin, anti-E-cadherin, anti-Vimentin (BD Biosciences, USA), anti-β-actin, anti-mouse and anti-rabbit secondary antibodies (Cell Signaling Technology).

### RNA interference

The small interfering RNA (siRNA) sequences corresponding to the target sequences and inhibitor of miR-let-7c were directly synthesized (GenePharma). The siRNA constructs were as follows: siRNA-circ_0039908-1: 5'-GUUUACUGUGAGAUAUCAATT-3' (sense) and 5'-UUGAUAUCUCACAGUAAACTT-3' (antisense), siRNA-circ_0039908-2: 5'-AUUGUUUACUGUGAGAUAUTT-3' (sense) and 5'-AUAUCUCACAGUAAACAAUTT-3' (antisense). Transfection of siRNA and inhibitor into cells were performed with Lipofectamine 2000 according to the instructions of the manufacturer.

### Cell proliferation analysis

Cell proliferation was evaluated using CCK-8 assays. Cells were digested and plated at a concentration of 3× 10^3^ cells per well in 96-well plates under standard cell culture conditions. A Cell Counting Kit-8 (Boster, Wuhan, China) was used to detect cell proliferation after culture for 24, 48, and 72h.

### Colony Formation Assay

A colony formation assay was used to confirm malignant transformation. Three thousand cells were seeded in 3ml of RPMI 1640 medium supplemented with 10% foetal bovine serum and incubated at 37 °C with 5% CO_2_. The number of colonies formed after 14 days was counted using ImageJ.

### Transwell assay

For the migration assay, cells were plated into the upper chamber of a Transwell insert in medium containing 1% FBS. The difference between the migration assay and invasion assay is that in invasion assay, the upper chamber is precoated with a Matrigel matrix (BD Science, Sparks, MD, USA). In the upper chamber, 3×10^4^ cells were seeded for the migration assay, while 5×10^4^ cells were seeded for the invasion assay. Then, 800 μl of complete medium was added to the lower chamber. After 24h of incubation, the cells that invaded from the upper surface to the lower chamber were fixed with methanol and stained with crystal violet. Finally, five random fields from each Transwell sample were selected to examine under a light microscope.

### Statistical analysis

We used Student's t-test (two-tailed) and two-way ANOVA to analyse the results when required. P<0.05 indicates that a difference between groups was significant. All data were analysed using GraphPad Prism 8.

## Results

### Identification of RNAs with differential expressions

**Figure 1:**
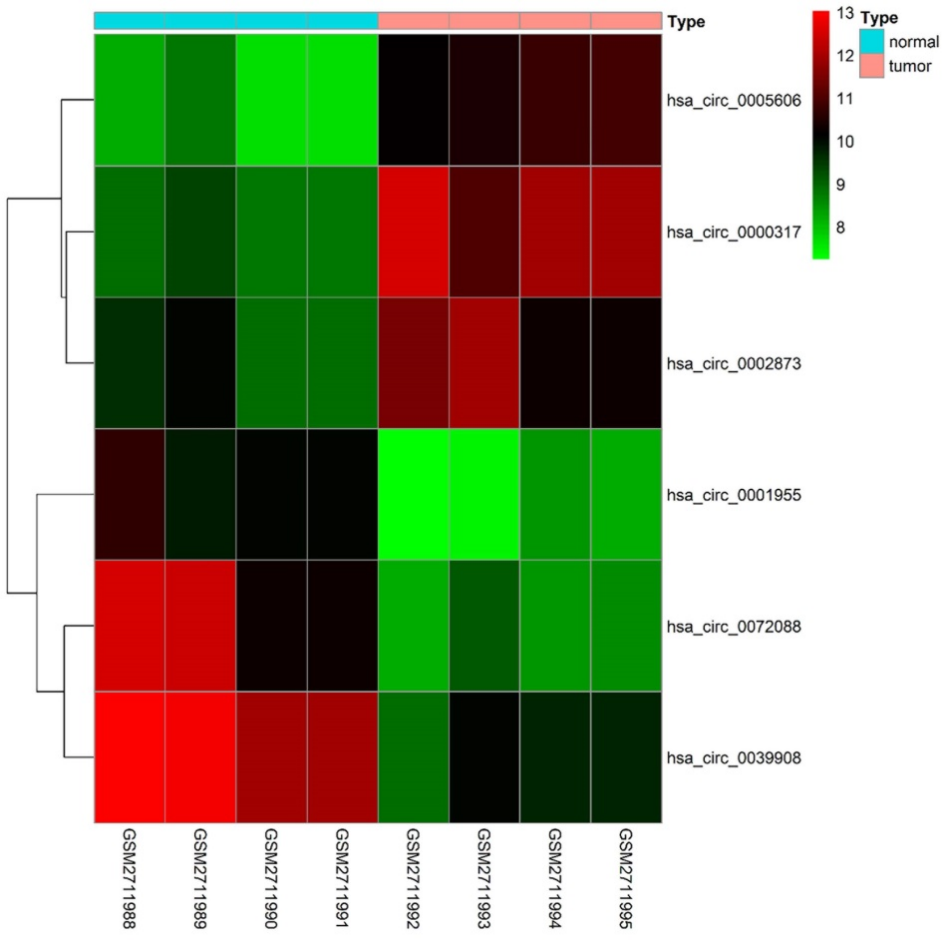
Heatmap of the differentially expressed circRNAs of the GSE101684 dataset.

**Figure 2:**
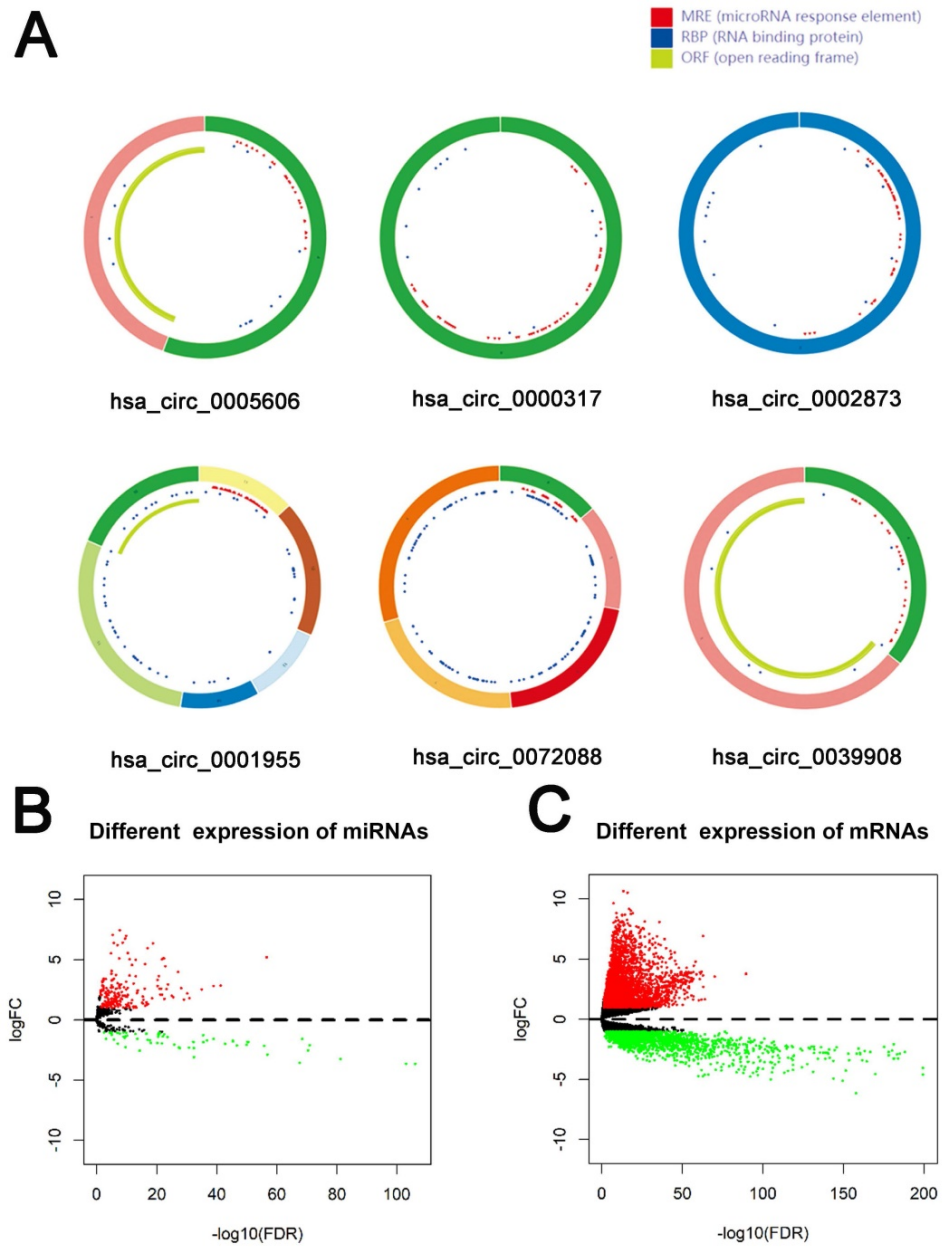
** Identification of RNAs with differential expressions.** (A) Schema graphs of 6 circRNAs downloaded from Cancer-Specific CircRNA (CSCD, http://gb.whu.edu.cn/CSCD/).Red spots represent miRNA response elements, blue spots represent RNA binding protein, and green curves represent open reading frame. (B, C) Volcano plot of differentially expressed miRNAs (B) and mRNAs (C).

79 differential circRNAs were screened, including 16 up-regulated circRNAs and 63 down-regulated circRNAs. 3 of the upregulated circRNAs and 3 downregulated circRNAs that can be retrieved in the CSCD database and were the most significant in expression difference were selected for further analysis. The expression of the 6 circRNAs were presented by heatmap (Fig. 1). Table 2 showed the primary features of the 6 circRNAs and Fig. 2A showed the fundamental modes of the structure. Through the analysis of DEmiRNAs and DEmRNAs obtained from the LUAD tissue samples and nearby healthy tissues in the TCGA database; we discovered 293 DEmiRNAs (226 exhibited up-regulation and 67 exhibited down-regulation, and 5598 DEmRNAs (3752 exhibited up-regulation and 1846 exhibited down-regulation) (Fig. 2B and 2C).

### Construction of the competing endogenous RNA network

**Figure 3:**
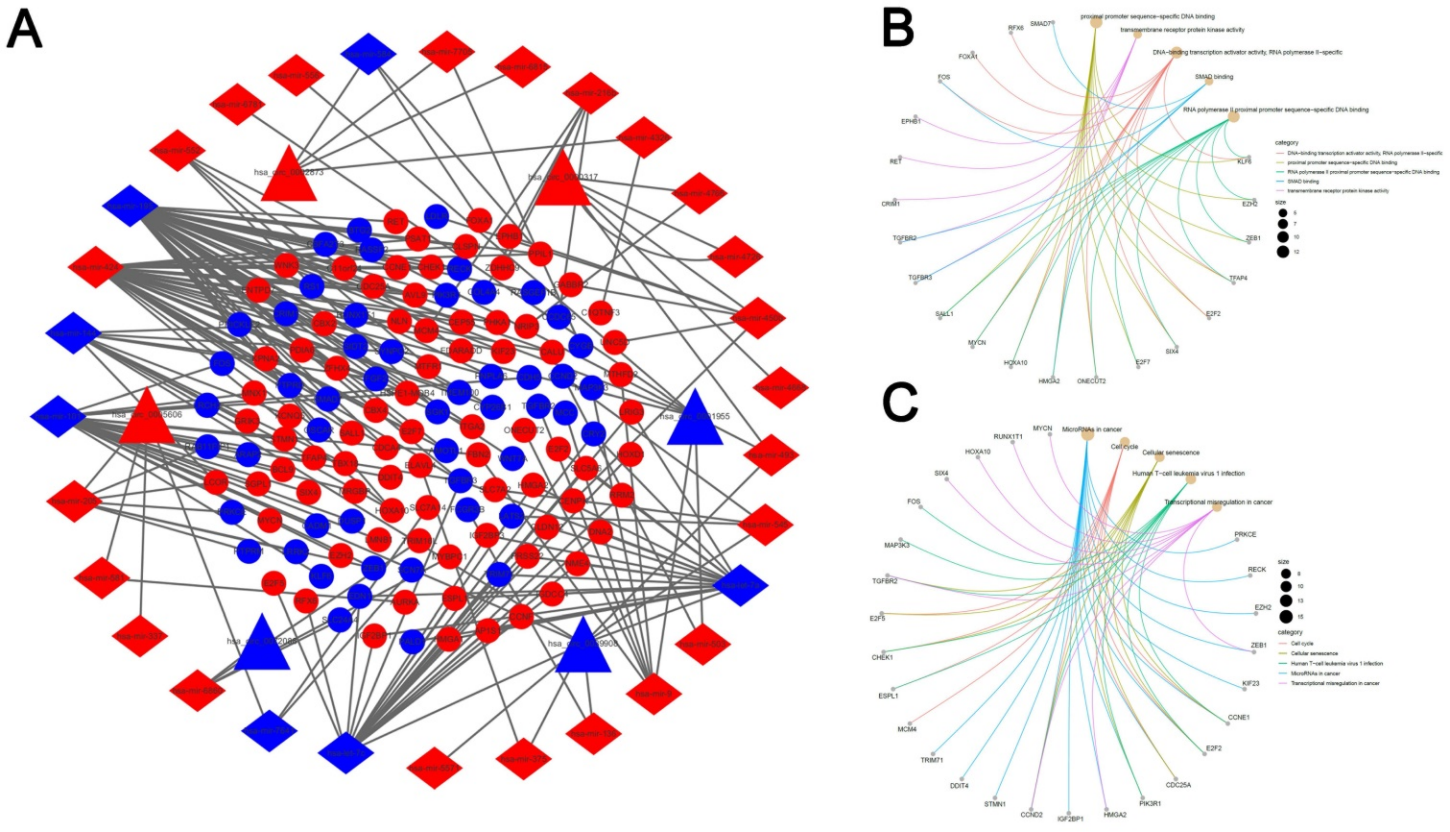
** Construction of the competing endogenous RNA network.** (A) The ceRNA network of circRNA-miRNA-mRNA in LUAD. The network consists of 6 circRNA nodes, 31 miRNA nodes, and 132 mRNA. (B, C) Cnet plot of the top 5 GO terms(A) and KEGG pathways(B) enriched by DEmRNA involved in the ceRNA network.

313 pairs of circRNA and corresponding miRNA were found by searching the 6 circRNAs in the CSCD database. We screened out 33 circRNA-miRNA pairs based on the intersection with the DEmiRNAs, which included 31 DEmiRNAs accompanied by 6 circRNAs. Additionally, through the prediction of three databases (miRDB, miRTarBase and TargetScan), we identified 1073 mRNAs. Next, we compared those mRNAs with the 5598 DEmRNAs and the precondition of being chosen as mRNA candidates were the overlapped recognition of all the three databases. It was suggested by the outcomes that 132 DEmRNAs were related to the network of ceRNA. Based on the nodes including 132 mRNAs, 31 miRNAs, as well as 6 circRNAs in LUAD, a network of ceRNA was established (Fig. 3A). Then the GO an KEGG analysis were performed by these mRNAs, and top 5 GO and KEGG terms (Fig. 3B, C).

### PPI network analyses

**Figure 4:**
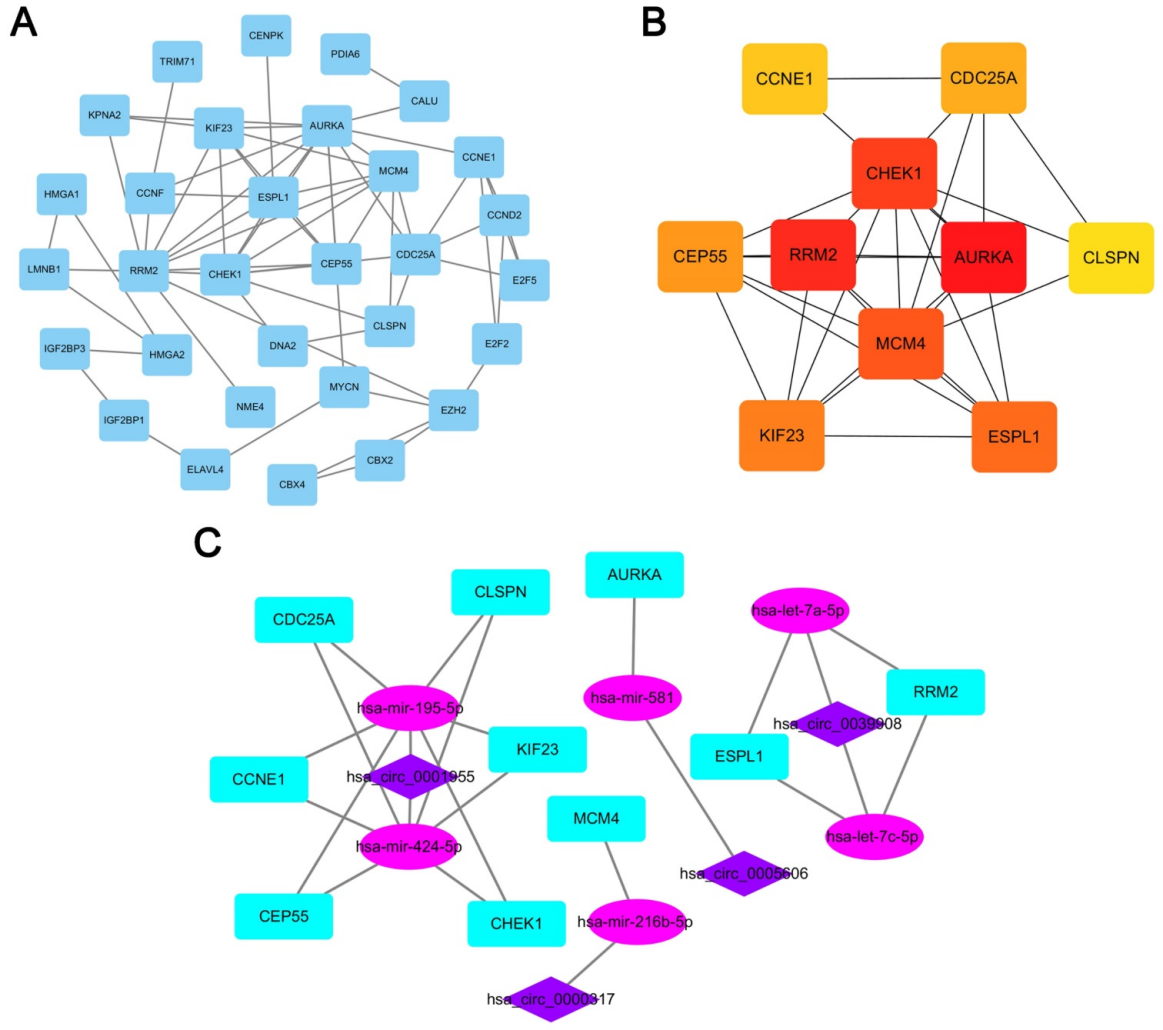
** PPI network analyses.** (A) PPI network of 132 genes, consisting of 31 nodes and 64 edges. (B) PPI network of 10 hubgenes. (C) CircRNA-miRNA-hubgene network. The network consists of 4 circRNAs, 6 miRNAs, and 10 hubgenes.

A PPI network consisting of 31 nodes and 64 edges was constructed using the STRING database, revealing the interrelationships of 132 DEmRNAs (Fig. 4A). For the identification of hub genes during the development of LUAD, the cytoHubba plugin was used to calculate the closeness centrality of DEmRNAs, which revealed 10 hub genes included CCNE1, CDC25A, CHEK1, CLSPN, AURKA, RRM2, MCM4, CEP55, KIF23 and ESPL1(Fig. 4B). According to the above hubgenes, a circRNA-miRNA-hubgene subnetwork was constructed (Fig. 4C), which contained 10 regulation modules of ceRNAs.

### Univariate and multivariate Cox regression analyses

**Figure 5:**
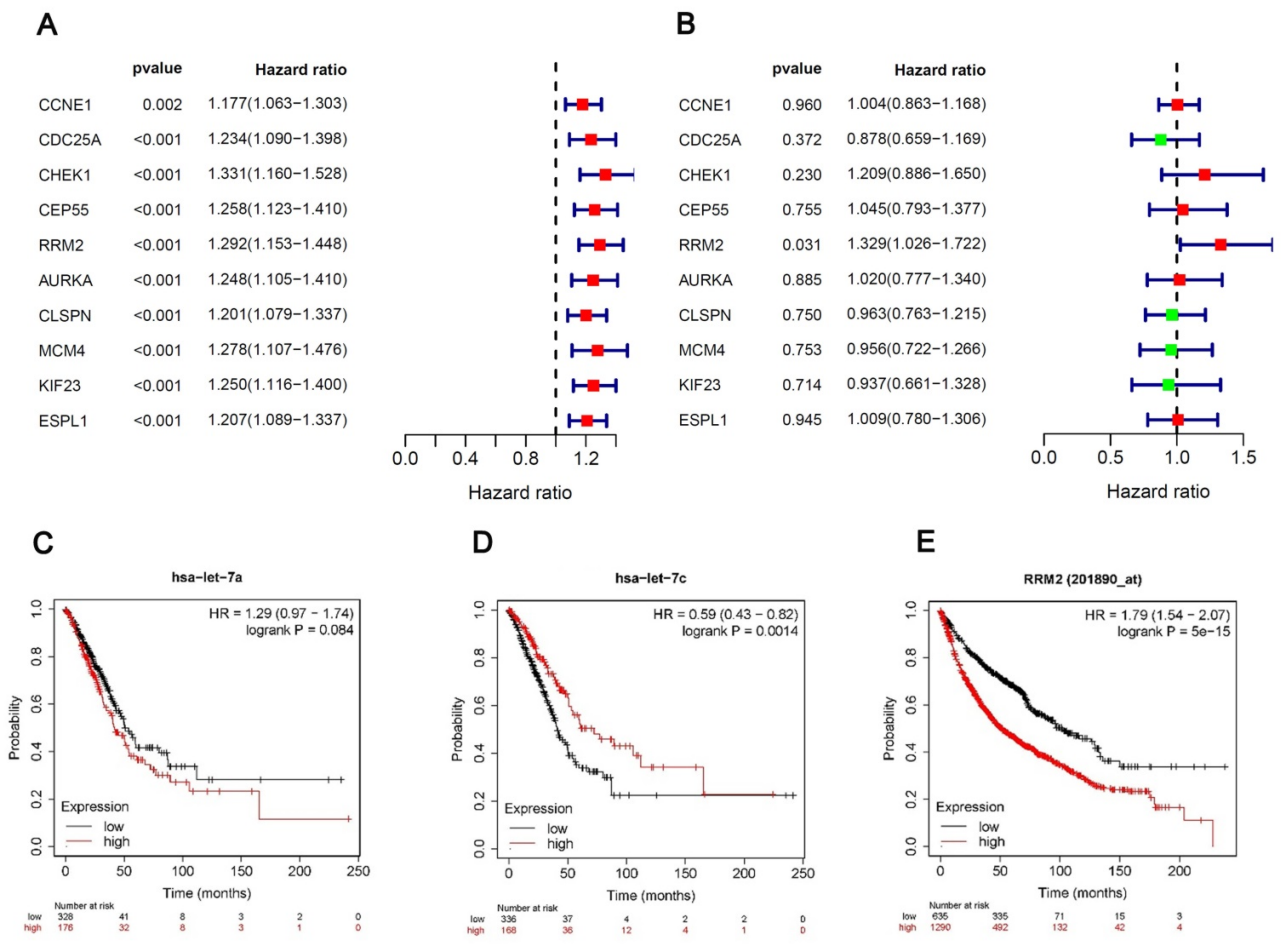
** Univariate and multivariate Cox regression analyses.** (A, B) Univariate and multivariate Cox regression analyses of the 10 hubgenes. (C-E) The KM plot of miR-let-7a-5p, miR-let-7c-5p and RRM2 in LUAD patients.

We performed univariate and multivariate Cox regression analyses of the 10 hubgenes, only the RRM2 had the significance in the results of both univariate and multivariate cox regression analyses (Fig. 5A, B), while the results of survival analysis revealed that the expression hsa-let-7c and RRM2 were significantly associated with the overall survival of patients (Fig. 5C-E), so we chose the circRNA_0039908/let7c-5p/RRM2 axis for further study.

### CircRNA_0039908 can regulate the expression of miR-let7c-5p and RRM2

**Figure 6:**
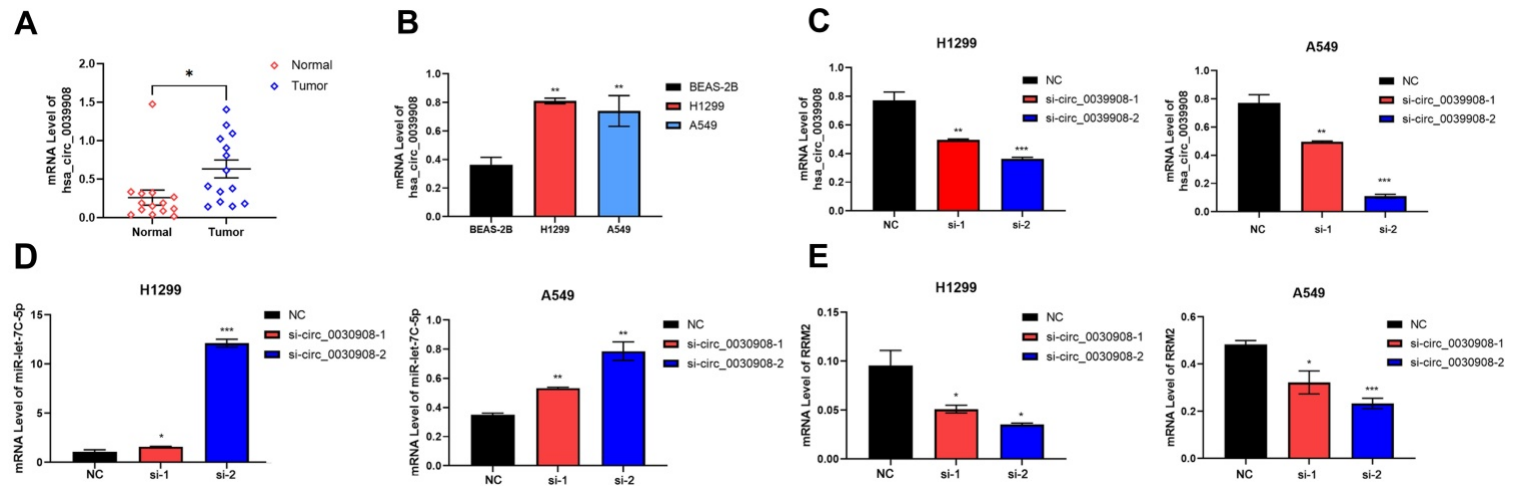
** CircRNA_0039908 can regulate the expression of miR-let7c-5p and RRM2.** (A) mRNA level of circ_0039908 in LUAD patients tissues. (B) Circ_0039908 was high-expressed in LUAD cells. (C) The knockdown efficiency of si- circ_0039908. (D, E) The expression of miR-let-7c and RRM2 after knockdown of circ_0039908. *P < 0.05; **P < 0.01; ***P < 0.001.

We collect 14 paired LUAD tissues and the corresponding adjacent tissues, the patients were diagnosed with LUAD in 2018-2021. qRT-PCR results indicated that circ_0039908 was over-expressed in LUAD tissues (Fig.6A), the same result can be also obtained in LUAD cells compared with that in human immortalized normal epithelial cells (Fig. 6B). Then we knockdown the circRNA_0039908 by siRNA (Fig. 6C) and we found that with the downregulation of circRNA_0039908, the expression of miR-let7c-5p was promoted (Fig. 6D)and the expression of RRM2 was inhibited (Fig. 6E). So, we identified that circRNA_0039908 can regulate the expression of miR-let7c-5p and RRM2.

### Knockdown circRNA_0039908 can inhibit the proliferation, invasion and migration of LUAD cells

**Figure 7:**
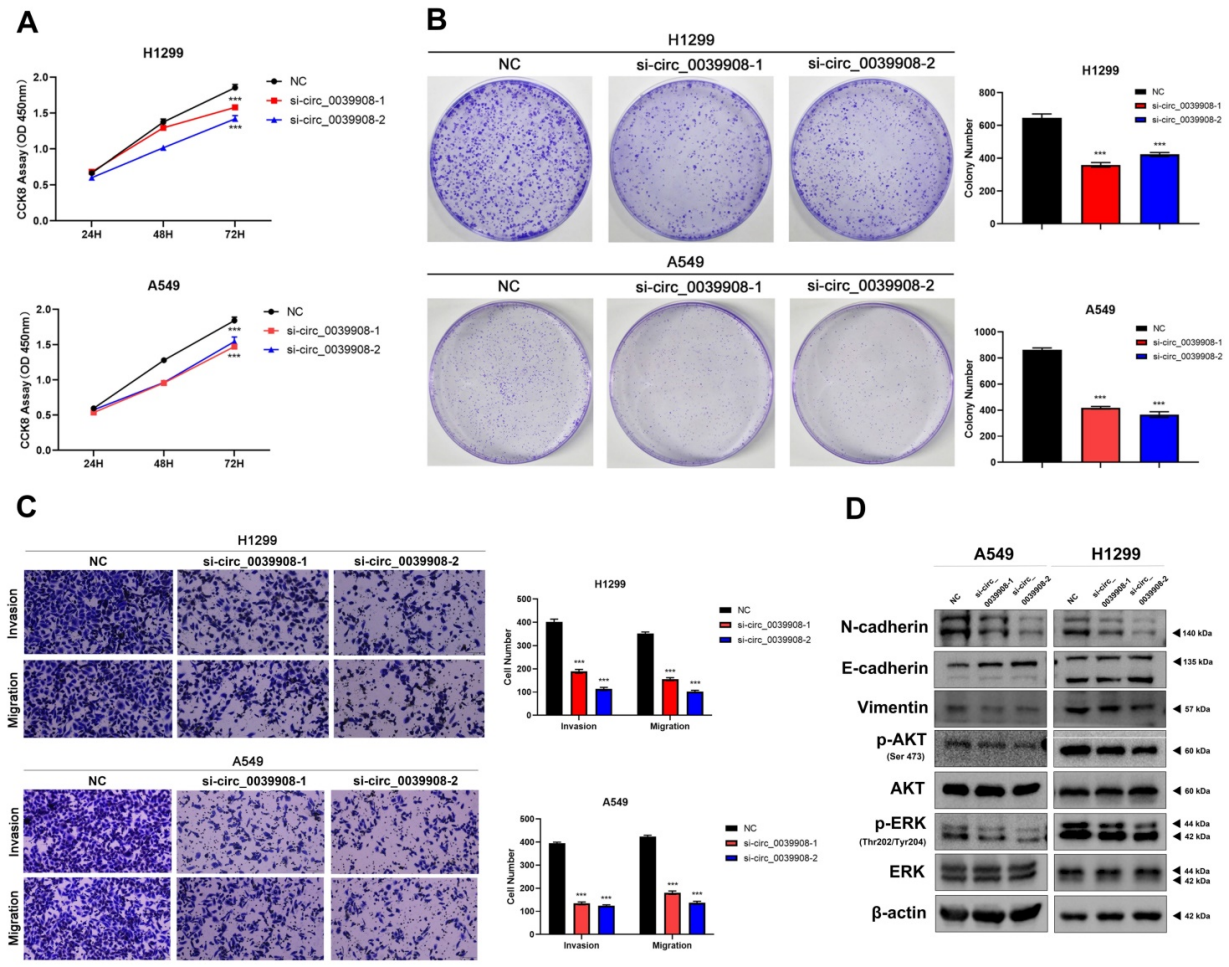
** Knockdown circRNA_0039908 can inhibit the proliferation, invasion and migration of LUAD cells.** (A) CCK-8 assay of cell viability in H1299 and A549 cells. (B) Representative images of the results of the clonogenic analysis of H1299 and A549 cells proliferation after transfection of si-circ_0039908. (C) Representative images of the transwell assay results for cell invasion and migration in H1299 and A549 cells after transfection of si-circ_0039908. (D)The protein expression of N-cadherin, E-cadherin, Vimentin, p-AKT, AKT, ERK, p-ERK, β-actin were evaluated by western blot.***P < 0.001.

**Figure 8:**
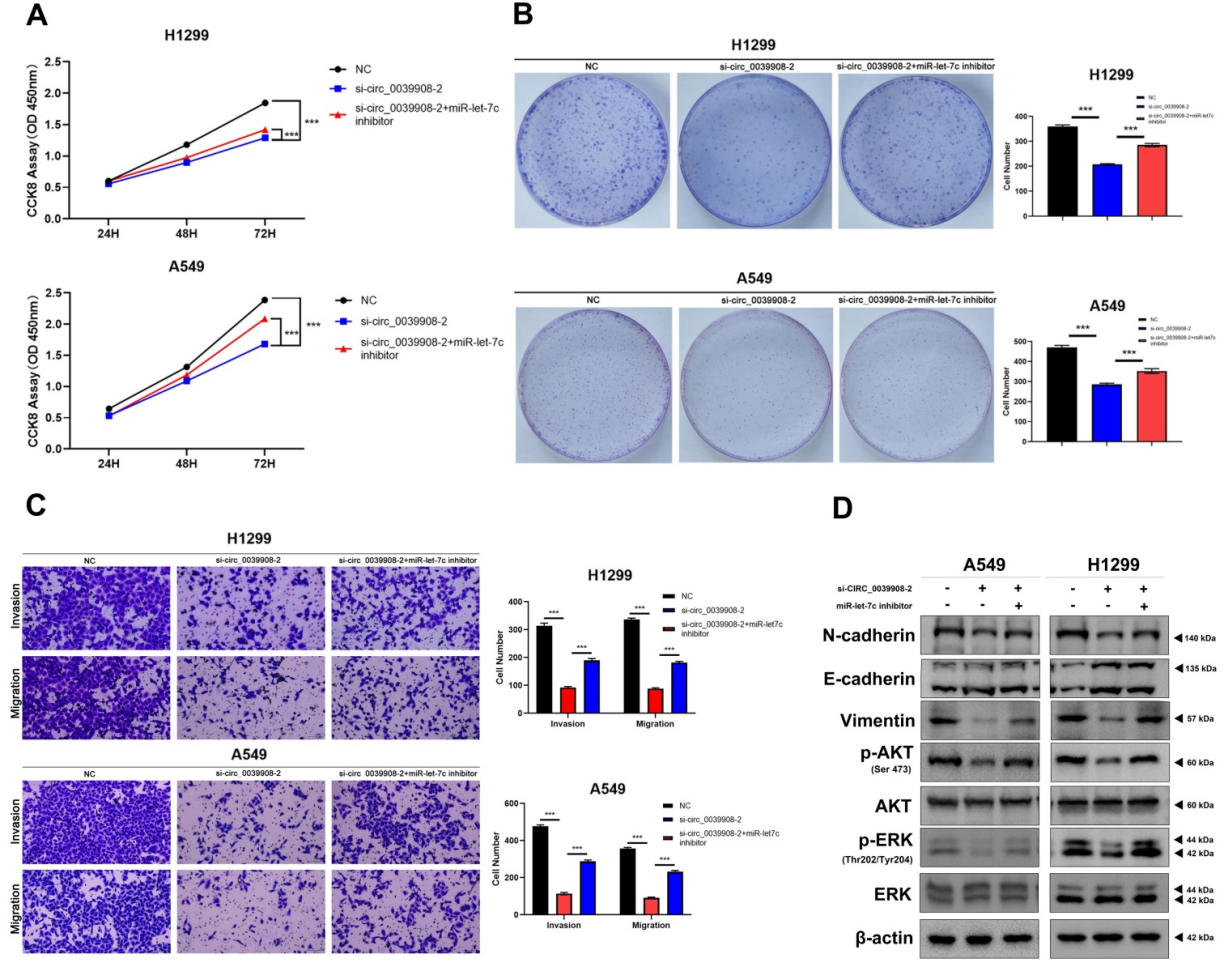
** Inhibition of miR-let-7c can rescure the effect of the knockdown of circ_0039908 in LUAD cells.** (A) CCK-8 assay of cell viability in H1299 and A549 cells after cells co-transfected with si-circ_0039908 and inhibitor of miR-let-7c. (B) Representative images of the results of the clonogenic analysis of H1299 and A549 cells. (C) Transwell assay results in H1299 and A549 cells. (D)The protein expression of N-cadherin, E-cadherin, Vimentin, p-AKT, AKT, ERK, p-ERK, β-actin were evaluated by western blot. ***P < 0.001.

To further characterize the role of circRNA_0039908 in LUAD cells, the CCK8 assay was performed to identify the effects of circRNA_0039908 on LUAD cells proliferation, we found that the inhibition of circRNA_0039908 can suppress the proliferation of LUAD cells (Fig. 7A), we also confirmed these results by colony formation assays (Fig. 7B). Transwell assays showed that knockdown of circRNA_0039908 can inhibit LUAD cells invasion and migration (Fig. 7C). Then western blot assays were performed to detected the protein expression after knockdown of circ_0039908, we found that the expression of p-AKT, p-ERK, N-cadherin and Vimentin can be inhibited, while E-cadherin was promoted after knockdown of circ_0039908 (Fig. 7D). The effects of knockdown of circRNA_0039908 can be reversed after the cells added with inhibitor of miR-let-7c (Fig. 8A-D).

## Discussion

In the past, circRNAs, considered as RNA transcriptional by-product, exhibited lower expression richness and no significant biological function 13. However, due to the development of bioinformatics and Next-Generation Sequencing technologies, the quantity of circRNAs identified in multiple species and verified as participating in varied vital pathophysiologic processes, which included tumorigenesis, exhibited an explosive growth 14-16. Moreover, since circRNAs are relatively stable, widespread expressed and presenting abundently in blood, saliva as well as exosomes, the detection is easy, which makes it an innovative and promising cancerous biomarker for diagnosis^6^, ^17^-^19^. However, it remains largely unclear about the precise effect exerted by circRNAs on LUAD. In our present research, we obtained circRNAs,miRNAs as well as mRNAs with differential expressions between LUAD tissue and non-tumor tissue samples from TCGA and GEO database, thereby constructing the network of circRNA-miRNA-mRNA regulation.

The mechanisms of circRNAs worked are multiple, CircRNAs in the nucleus can regulate transcription, splicing and chromatin interactions 20. Once in the cytoplasm, some circRNAs function as competing endogenous RNAs (ceRNAs), which are defined as miRNA Intragenic antagonistic roles of protein and circRNA in tumorigenesis 21. CircRNA can also function as protein scaffolds, exon-intron- containing circRNAs can promote transcription of their parent genes by interacting with U1 small nuclear ribonucleoproteins and with Pol II at the promoters of their parent genes 22. Some circRNAs can sequester proteins, by promoting the generation of circMbl, increased expression of the multifunctional protein MBL leads to reduced production of the linear Mbl mRNA; in turn, circMbl sequesters MBL and prevents it from carrying out other neural functions 23.

Multiple researches demonstrated the circRNA expressions in LUAD to be dysregulated and to be associated with aggressive clinicopathological features and dismal outcome. Wang et al analyzed thirty‐six paired LUAD and healthy tissue samples and discovered the negative association between cMras expression and tumorous stages. Furthermore, they confirmed the inhibiting effect of cMras on tumor metastasis and development. As for LUAD, cMras restrained the activity of miRNA-567 directly like a sponge, thereby upregulating the target gene PTPRG which acted as a tumor-inhibiting factor 24. Zhou et al revealed the regulatory effect of circRNA on glycolysis in LUAD and proved the promoting role exerted by circRNA-ENO1 in the EMT and proliferation of LUAD through the upregulation of the host gene ENO1, which made circRNA-ENO1 an innovative and promising biomarker for lung cancer 25. Ying et al certified that hsa_circ_0000317, hsa_circ_0005606, hsa_circ_0002873, hsa_circ_0001955, hsa_circ_0072088 and hsa_circ_0039908 were dysregulated in LUAD tissues. Among them, hsa_circ_0072088 was found to participate in the regulation of cell migration and proliferation of colorectal carcinoma through the miR532e3P/FOXO4 axis 26. In addition, hsa_circ_0001955 has been investigated to be overexpressed in breast carcinoma which could be involved in BC-triggering biological processes and pathways 27. It was also demonstrated that hsa_circ_0001955 could play an oncogenic role in hepatocellular carcinoma through the hsa_circ_0001955/miR-145-5p/NRAS axis 28. Nevertheless, no one has proposed the role of any of the other 4 circRNAs in tumors yet. MiRNAs refer to a kind of non-coding RNAs with high conservation containing about 22 nucleotides 28. They can interact with mRNAs-coding genes by binding to their 3'untranslated regions (UTRs) for directly post-transcriptional regulation of the expression of target genes 29. MiRNAs can regulate approximately 60% of human genes and exert an essential role in nearly every regard of oncology, which includes the proliferating, apoptotic, invading, metastatic and angiogenic processes 30. In the current work, 31 DEmiRNAs were identified from the network of ceRNAs, in which a portion had been proved to be essential to the processes of LUAD generation and progression. MiRNA-let-7c-5p, the miRNA studied in our research, has been widely reported in different cancer types, it can mediate the pathogenesis progression of NSCLC by targeting ITGB3 and MAP4K3 31, and promote the sensitivity of LUAD cells to EGFR-TKIs by inhibiting the WNT pathway 32.

Ribonucleoside-diphosphate reductase subunit M2, also known as ribonucleotide reductase small subunit, is an enzyme that in humans is encoded by the RRM2 gene, inhibition of RRM2 can promote the apoptosis of NSCLC and head and neck squamous cell carcinoma (HNSCC) cell lines by increasing Bcl-2 degradation 33. While it can mediate NSCLC pathogenesis by regulating PI3K/Akt and WNT pathway 34, 35. RRM2 could be regulated by multiple non-coding RNAs, just like miR-20a-5p, miR520a and miR-143-3p 34-36.

## Conclusion

In conclusion, we screened out that circ_0039908 was high-expressed in LUAD by bioinformatics, while inhibition of circ_0039908 can suppress the proliferation, invasion and migration of LUAD cells, then we found that circ_0039908 affected the pathogenesis of LUAD cells by regulating miR-let-7c/RRM2, so the circ_0039908/miR-let-7c/RRM2 axis was identified played an important role in LUAD and it may provide us with a new insight in diagnosis and therapeutic methods of LUAD patients.

## Figures and Tables

**Table 1:** Primers sequences used in the study

	Primer Sequences
circ_0039908	F: GGACATTGTTTACTGTGAGATATCA R: ATTACTTTGATATATGTGTTCTGGC
miR-let-7c	F: TGAGGTAGTAGGTTGTATAGTT
RRM2	F: CACGGAGCCGAAAACTAAAGC
	R: TCTGCCTTCTTATACATCTGCCA
β-actin	F: CACAGAGCCTCGCCTTTGC
	R: ACCCATGCCCACCATCACG
U6	F: GGAACGATACAGAGAAGATTAGC
	R: TGGAACGCTTCACGAATTTGCG

**Table 2:** Basic characteristics of the 6 differently expressed circRNAs.

circRNA ID	position	genomic length	strand	gene symbol	best transcript	regulation
hsa_circ_0000317	chr11:62288378-62288522	144	-	AHNAK	NM_001620	up
hsa_circ_0005606	chr16:1675973-1682366	6393	+	CRAMP1L	NM_020825	up
hsa_circ_0002873	chr12:12788710-12788908	198	+	CREBL2	NM_001310	up
hsa_circ_0001955	chr15:64495280-64508912	13632	-	CSNK1G1	NM_022048	down
hsa_circ_0072088	chr5:32379220-32388780	9560	-	ZFR	NM_016107	down
hsa_circ_0039908	chr16:68059317-68072052	12735	+	DUS2L	NM_017803	down

**Table 3:** Detailed clinic parameters of enrolled patients

Gender	Age	Stage	Smoker
Male	49	T1N0M0	No
Male	64	T1N0M0	No
Female	65	T1N0M0	YES
Female	65	T1N1M0	YES
Male	63	T1N0M0	No
Female	57	T1N0M0	No
Male	51	T2N2M0	No
Male	60	T1N0M0	No
Female	74	T1N0M0	YES
Female	69	T1N0M0	YES
Male	69	T1N0M0	No
Male	72	T2N0M0	No
Female	55	T2N0M0	YES
Female	74	T1N0M0	YES

## Abbreviations

CCK8: Cell Counting Kit-8
LUAD: Lung adenocarcinoma
NSCLC: Non-small cell lung cancer
TCGA: The Cancer Genome Atlas
GEO: Gene Expression Omnibus
ceRNA: Competing Endogenous RNA
